# Perceived discrimination and depression among low-income Latina male-to-female transgender women

**DOI:** 10.1186/1471-2458-12-663

**Published:** 2012-08-15

**Authors:** Mohsen Bazargan, Frank Galvan

**Affiliations:** 1 Professor and Director of Research, Department of Family Medicine and Public Health Program, Charles R. Drew University of Medicine and Science, 1731 East 120th Street, Los Angeles, CA 90059, USA; 2 Director of Research and Evaluation, Bienestar Human Services, Inc, 5326 East Beverly Blvd, Los Angeles, CA 90022, USA

## Abstract

**Background:**

This study examines exposure to perceived discrimination and its association with depression among low-income, Latina male-to-female transgender women as well as evaluates the impact of sexual partner violence and mistreatment on depression.

**Methods:**

A total of 220 Latina male-to-female transgender women who resided in Los Angeles, California, were recruited through community based organizations and referrals. Participants completed individual interviews using a structured questionnaire. Depressive symptoms were assessed using the Patient Health Questionnaire (PHQ-9). Perceived discrimination was assessed using a fifteen-item measure that was designed to assess the experiences of maltreatment of transgender individuals. Multinomial logistic regression was used to examine the association between perceived discrimination and depression after controlling for the presence of other variables.

**Results:**

Of the sample, 35% reported significant depressive symptoms (PHQ-9 ≥ 15). Additionally, one-third of the participants indicated that in the two weeks prior to the interviews they had thought either of hurting themselves or that they would be better off dead. The extent of perceived discrimination in this population was extensive. Many of the participants experienced discrimination on a daily basis (14%) or at least once or twice a week (25%) as demonstrated by a positive response to at least 7 of 15 items in the measure of perceived discrimination. Almost six out of ten participants admitted that they had been victims of sexual partner violence. Those who reported more frequent discrimination were more likely to be identified with severe depression. There was also a notable association between self-reported history of sexual partner violence and depression severity.

**Conclusions:**

A significant association between depression severity and perceived discrimination was identified. How exposure to discrimination leads to increased risk of mental health problems needs additional investigation. Models investigating the association between perceived discrimination and depression among transgender women should include sexual partner violence as a potential confounding variable.

## Background

Mental health disorders remain one of the most significant areas of clinical concern for lesbian, gay, bisexual, and transgender (LGBT) individuals [1]. In LGBT communities, male-to-female transgender individuals are thought to be at the highest risk for mental health problems. Depression [2-6] and suicidal thoughts [3,5-8] are the two most common mental health problems, which may be caused by these individuals’ negative social encounters with others [9,10], gender-related physical and psychological abuse and violence, [6-8] social isolation [2,6,8] or a need to hide their transgender life from others [2]. However, a review of the current literature clearly shows that transgender people’s mental health issues have been neglected by researchers [3,11,12]. A recent content analysis of articles published in 17 marriage/couple and family therapy journals from 1997 through 2009 support this assertion that transgender issues are ignored and marginalized by scholars and researchers [13] .

According to a recent report of the Task Force on Gender Identity and Gender Variance conducted by the American Psychological Association, transgender people experience a wide range of psychological challenges for which mental health care may be of benefit [14]. These include gender identity distress, stigma, bias, discrimination, and lack of social support [12]. However, many transgender people suffering from problems related to these issues may be reluctant to seek mental health care because mental health professionals’ lack of training and basic cultural competency [15] or bias toward transgender clients [16].

Transgender individuals are more likely to experience discrimination, stigmatization, victimization, and stressful life events compared to gay men, lesbians, and bisexuals [17,18]. Furthermore, transgender individuals who are also ethnic minorities are even more likely to be subjected to excessive discrimination and stigmatization associated with both racism and heterosexism [6,19]. However, there is limited information on the association between the mental health status of Latina transgender women and the pressures and stress associated with discrimination related to being transgender (transphobia). This is important because several studies have reported a pervasive pattern of economic, health care, housing and career and employment discrimination and prejudice against LGBT people within society [5,11,18,20-29].

This study examines perceived discrimination related to being transgender and its association with depression among low-income, Latina male-to-female transgender women as well as evaluates the impact of sexual partner violence and mistreatment on depression. Although sexual partner violence is also found among this segment of this population [30,31], similar to physical and mental health-related issues, the medical community has often neglected to address transgender sexual partner violence [32].

## Methods

### Sample and recruitment

The study sample was composed of 220 low-income Latina male-to-female transgender women, ages 18 years and older, living in Los Angeles, California. The participants were recruited through community based organizations, support groups, social events, community outreach, and personal referrals by other transgender individuals. The face-to-face surveys were conducted over a six month period. Almost all of the surveys (215 out of 220) were conducted in Spanish, according to the subject’s preference. Each interview lasted approximately 30–45 minutes and was conducted in a private room at a community based organization (CBO). Following the completion of the survey, participants received a gift card worth $20. Only three individuals who were approached refused to participate in this study. Participation in this study was entirely voluntary and was sought only after written consent was obtained. During the pilot testing for this study with a small number of transgender individuals, it was suggested by the participants that $20 would be an appropriate remuneration for this level of data collection. This study was reviewed and approved by the Institutional Review Board of Charles R. Drew University of Medicine and Science.

### Measurement

The survey instrument was a collection of internally developed questions and validated instruments taken from various sources [33,34]. The survey was pilot tested with a small number of transgender volunteers. Appropriate modifications were made based on the pilot test, cognitive interviews and expert reviews of the instruments by two medical sociologists, a health service researcher and two social workers.

Depression severity was measured using a Spanish language version of the Patient Health Questionnaire-9 (PHQ-9). The PHQ-9 is an assessment tool developed to diagnose the presence and severity of depression in primary care. The nine items of the PHQ-9 relate to the most common and sensitive symptoms in depression. Each of the Diagnostic and Statistical Manual of Mental Disorders (DSM-IV) [35] symptoms of depression are assessed by asking the individual to recall their frequency over a 2-week period as “0” (Not at all), “1” (Several Days), “2” (More than Half the Days) or “3” (Nearly Every Day). The PHQ-9 has the potential of being a dual-purpose instrument that, with the same nine items, can suggest a depressive disorder diagnosis using a categorical algorithm and also grade the depressive symptom severity. As a severity measure, the scores can range from 0 to 27. Scores of 0–4 indicate no - minimal depression, 5–9 indicate mild depression, 10–14 indicate moderate depression requiring a treatment plan and/or counseling, 15–19 indicate moderately severe depression requiring active treatment and 20–27 indicate severe depression requiring immediate pharmacotherapy and counseling with a mental health specialist [36]. The PHQ-9 has demonstrated acceptable reliability, validity, sensitivity, and specificity. Specifically, in a 2007 meta-analysis of the questionnaire, a PHQ-9 score greater that 10 had a pooled sensitivity of 80% and a specificity of 92% for major depression [33].

Recent studies examining the psychometric properties of the Spanish language version of this instrument suggest that the PHQ-9 can be used with confidence in both English and Spanish versions to screen Latinas for depression [37]. Similar to the alpha reliability scores for both the English and Spanish versions of the PHQ-9 found in that study, the alpha reliability of this scale for the present sample was 0.89.

For purposes of evaluating the impact of independent variables on different levels of depression severity, three groups of depression severity were created. The “Low Severity” category was created to indicate no depression to low depression severity and was defined as PHQ-9 scores of 0–9. This combines the PHQ-9 categories of None - minimal and Mild. The “Moderate” category coincides with the PHQ-9 scoring category of the same name and indicates a moderate depression severity. This is defined by PHQ-9 scores of 10–14. The “High Severity” category was created by combining the PHQ-9 scoring categories of Moderately Severe and Severe. This category is the highest depression severity and was defined as PHQ-9 scores 15–27 [36].

Perceived discrimination was assessed using a fifteen-item measure that focuses on experiences of maltreatment that are relatively common. The scale was redesigned to assess experiences of maltreatment specific to transgender individuals. Several items used in this scale are similar to items that have been employed by Williams and his colleagues [34] to measure “perceived everyday racial and ethnic discrimination” in minority populations. Some sample questions included the following:

1. How often have you been treated with less courtesy than other people for being transgender?

2. How often have you received poorer service than other people at restaurants or stores for being transgender?

3. How often have you been called names or insulted for being transgender?

The frequency of each experience was rated on a 4-point scale (4 = often, 3 = sometimes, 2 = rarely, and 1 = never). The internal consistency estimate (Cronbach’s alpha) for the overall summated 15-item scale was very high (0.87), and 50% of the correlation coefficients among the 15-items were greater than 0.35. The total score ranged from 15 to 60 (33.4 ± 9.3), and higher scores on this index represented a higher level of perceived discrimination.

Sexual partner violence and mistreatment by sexual partners or spouse were assessed using two items: 1) Have you ever been a victim of domestic violence by your partner/spouse?; and 2) Have you ever been mistreated by a main or casual partner? The index ranged from zero to two, where scores of zero and two indicate that participants had reported none or both domestic violence and mistreatment, respectively. A score of one shows that participants had reported domestic violence or mistreatment.

History of sex work was measured by asking participants 5 questions which allowed them to report if they had ever exchanged sex for money, shelter, food, drugs, or anything else. This item was scored “Yes” if the participant answered affirmatively to any item.

Other measures collected included demographic data, particularly age, education, immigration status, years living in United States, living arrangement, income and perceived rejection by family members.

### Statistical Analysis

The statistical analysis was performed with the SPSS® program (SPSS 19.0 for Windows, SPSS Inc., Chicago, IL, USA). In the analyses, participants’ demographic characteristics including age, education, income, immigration status, living arrangement, and years living in USA were controlled. In addition, history of sex work was included in the data analysis [12]. Other independent variables in this research included perceived discrimination and history of abuse and mistreatment. In addition to a descriptive analysis of all variables, bivariate chi square and ANOVA were conducted to determine the relationships between each of the depression severity groups and the independent variables mentioned above. In addition, multinomial logistic regression was applied for studying the effect of perceived discrimination on depression after controlling for other variables. We utilized a p-value < 0.05 to identify statistically significant differences. To avoid multicollinearity, a diagnostic test was performed in the multivariate analysis to examine inter-correlation among independent variables. The largest relationship between independent variables was 0.37 (years lived in the United States and age).

## Results

Overall, in the sample of 220 participants aged 19 years and older, 157 (71%) were born in Mexico and 63 (29%) in other Latin American countries. The mean age of the sample was 36 years old (SD = 9), with a range of 19 to 57 years of age. Almost 87% of these participants had lived in the United States for more than five years (Table 1). Forty seven percent of the respondents admitted that they were undocumented. Forty four percent of the subjects indicated that they had no formal education beyond the 11th grade. Over 42% reported living alone. Fifty two percent of the sample reported either full-time (24%) or part-time (28%) employment status. While 52% of participants reported that their annual family income was less than $10,000, only 16% of participants reported an annual income of their household of more than $20,000. Twenty-eight percent of respondents reported having some type of insurance coverage.

**Table 1:** Characteristics of Study Sample and Bivariate Analysis of Correlates of Depression among Latina Male-to-Female Transgender Women(n = 220)

**Independent Variables**	**Total N (%) [Mean ± SD]**	**Depression (PHQ-9)**			**p value**
		**Low Severity N (%)[Mean ± SD]**	**Moderate Severity N(%)[Mean ± SD]**	**High Severity N (%)[Mean ± SD]**	
**Age**					0.426
< 35	100 (45)	66 (68)	17 (17)	15 (15)	
≥ 35	120 (55)	77 (64)	17 (14)	26 (22)	
**Education**					0 .002
< 12^th^ grade	96 (44)	56 (58)	12 (13)	28 (29)	
≥ 12^th^ grade	124 (56)	89 (72)	22 (18)	13 (11)	
**Immigration Status**					0.998
Legal Resident	117 (53)	77 (66)	18 (15)	22 (19)	
Undocumented	103 (47)	68 (66)	16 (16)	19 (18)	
**Years in USA**					0.073
< 5 years	29 (13)	22 (76)	6 (21)	1 (3)	
≥ 5 years	191 (87)	123 (64)	28 (15)	40 (21)	
**Living Arrangement**					0.956
Alone	92 (42)	60 (65)	14 (15)	18 (20)	
With other	128 (58)	85 (66)	20 (16)	23 (18)	
**Income**					0.357
< $10,000	115 (52)	71 (62)	19 (17)	25 (22)	
≥ $10,000	105 (48)	74 (71)	15 (14)	16 (15)	
**History of Sex Work**					
No	44 (20)	26 (59)	7 (16)	11 (25)	0.451
Yes	176 (80)	119 (68)	27 (15)	30 (17)	
**Sexual Partner Violence **(Range 0–2)	[1.12 ± 0.82]	**[0.99 ± 0.81]**	**[1.24 ± 0.85]**	**[1.46 ± 0.74]**	0.003
**Perceived Discrimination** (Range 15–60: Low to High)	[42 ± 9.6]	**[31.4 ± 9.0]**	**[35.6 ± 9.7]**	**[39.0 ± 9.2]**	0.001

Low Severity is PHQ-9 scores of 0–9. Moderate Severity is PHQ-9 scores of 10–14. High Severity is PHQ-9 scores of 15–27.

Only 20% of the participants had never exchanged sex for money, drugs, shelter, or other things. Forty-five percent of them currently were engaged in sex-work. A large number of participants reported that they had been a victim of domestic violence by their partners or spouse (57%) or mistreatment by a main or casual partner (55%). Of those who reported being mistreated by their main or causal partners, 74% admitted that they had been a victim of domestic violence by their partners or spouse. However, 28% of those who had been a victim of domestic violence by their partners or spouse did not reported to mistreatment by their main or casual partner.

The extent of perceived discrimination in this population was extensive (Table 2). Many of the participants perceived discrimination on a daily basis (14%) or at least once or twice a week (25%) as demonstrated by a positive response to at least 7 of 15 items in the measure of perceived discrimination. Twenty six percent of the participants reported that they were harassed at least once or twice a week. Thirty three percent of participants indicated that people thought they had a mental problem because they are transgender. Thirty percent of the participants said that they were made fun of every day for being transgender. Twenty eight percent of participants were called names or insulted on a daily basis. Additionally, the data showed that almost 60% of the sample reported having been a victim of violence by their partner, spouse or casual partner and 66% of participants reported that they have been rejected by their family.

**Table 2:** Perceived Discrimination (n = 220)

**Perceived Discrimination**	**Never**	**Couple Times a Month**	**Once or Twice a Week**	**Daily**
1. How often have you been treated with less courtesy than other people for being transgender?	51 (23%)	107 (49%)	37 (17%)	25 (11%)
2. How often have you been treated with less respect than other people for being transgender?	35 (16%)	115 (52%)	42 (19%)	28 (13%)
3. How often have you received poorer service than other people at restaurants or stores for being transgender?	92 (42%)	91 (41%)	27 (12%)	10 (5%)
4. How often have people acted as if they are afraid of you for being transgender?	82 (37%)	94 (43%)	24 (11%)	20 (9%)
5. How often have you been called names or insulted for being transgender?	27 (12%)	95 (43%)	36 (16%)	62 (28%)
6. How often have you been threatened or harassed for being transgender?	61 (28%)	103 (47%)	23 (11%)	33 (15%)
7. How often have you been followed around in stores, the street or other places for being transgender?	70 (32%)	87 (40%)	33 (15%)	30 (14%)
8. How often have you been made fun of for being transgender?	32 (15%)	90 (41%)	32 (15%)	66 (30%)
9. How often have you been physically assaulted for being transgender?	107 (49%)	90 (41%)	9 (4%)	14 (6%)
10. How often do people think that you were a sex worker for being transgender?	42 (19%)	62 (28%)	23 (11%)	93 (42%)
11. How often do people think that you have a mental problem because you are transgender?	72 (33%)	59 (27%)	16 (7%)	73 (33%)
12. How often do people think that you abuse street drugs or alcohol because you are transgender?	90 (41%)	53 (24%)	19 (%)	58 (26%)
13. How often do people think that you are HIV positive or have an STI because you are transgender?	58 (26%)	75 (34%)	15 (7%)	72 (33%)
14. How often were you in a situation that you thought people felt sorry for you?	80 (36%)	88 (40%)	17 (8%)	35 (16%)
15. How often do you feel that people around you are uncomfortable because you are transgender?	54 (25%)	95 (43%)	25 (11%)	46 (21%)

About 35% of participants were identified with a moderately severe to severe level of depression (PHQ-9 ≥ 15). Sixty-five percent of participants had a depression score greater than 9 (minimal symptoms) or 10–14 (moderate symptoms).

### Bivariate and multivariate analysis

Table 1 reports demographic and socio-psychological characteristics of the sample and bivariate correlates of severity of depression. Among the demographic characteristics only education was significantly associated with severity of depression. Participants who had not completed high school were more likely to report depression than those with more education (p < .05). Additionally, at the bivariate level, a higher level of perceived discrimination and sexual partner violence and mistreatment were both associated with a higher level of depression.

Table 3 reports the regression estimates of the effects of the independent variables on the level of depression severity. Participants who reported a higher level of perceived discrimination were more likely to be identified with moderately severe to severe levels of depression (OR = 1.2; p < 0.01). Participants who reported experiencing sexual partner violence and mistreatment were 1.91 times more likely than their counterparts (who did not report sexual partner violence or mistreatment) to be identified with moderately severe to severe levels of depression (p < 0.01). Finally, participants who indicated that they had no formal education beyond the 11th grade (OR = 4.14; p < 0.01) were more likely to be identified with moderately severe to severe levels of depression.

**Table 3:** Multinomial Logistic Regression between Independent Variables and Depression among Latina Male-to-Female Transgender Women (n = 220)

**Independent variables**	**Moderate**		**Moderately severe to severe**	
	**Odds ratio**	**CI**	**Odds ratio**	**CI**
**Age**				
< 35	1.13	0.48 – 2.68	1.01	0.42 – 2.43
≥ 35	1.00	-	1.00	-
**Education**				
< 12^th^ grade	0.90	0.39 – 2.04	4.14**	**1.76 – 8.77**
≥ 12^th^ grade	1.00	-	1.00	-
**Immigration Status**				
Legal Resident	0.82	0.35 – 1.91	0.83	0.35 – 1.94
Undocumented	1.00	-	1.00	-
**Years in USA**				
< 5 years	1.22	0.39 – 3.83	0.11	0.01 – 1.00
≥ 5 years	1.00	-	1.00	-
**Living Arrangement**				
Alone	1.07	0.47 – 2.43	1.05	0.45 – 2.45
With other	1.00	-	1.00	-
**Income**				
< $10,000	1.32	0.57 – 3.05	1.23	0.52 – 2.92
≥ $10,000	1.00	-	1.00	-
**History of Sex Work**				
No	1.24	0.46 – 3.30	1.88	0.70 – 5.05
Yes	1.00	-	1.00	-
**Sexual Partner Violence (Range 0–2)**	1.29	0.79– 2.10	1.91**	**1.12– 3.26**
**Perceived Discrimination (Range 15 – 60: Low to High)**	1.05*	**1.01 – 1.10**	1.20**	**1.11 – 1.29**

The reference category is “minimal symptoms or minor depression” = 1.

The Chi-Square statistics is the difference in −2 log-likelihoods between the final model and a reduced model. The reduced model is formed by omitting an effect from the final model.

CI indicates 95% Confidence Interval for Odds Ratio.

Nagelkerke R-Squared = 0.252.

−2 Log Likelihood = 384.

Chi-Square for overall model with df 18 = 51.4 (p < 0.0001).

** P < 0.01; * P < 0.05.

## Discussion

This study examined the association between perceived discrimination and depression in low-income, Latina, male-to-female transgender women. Similar to previous studies, a large number of transgender participants in this study suffered from depression [2-4,6,38,39]. Thirty-five percent of the participants met the criteria for depression, compared to the 6.4% of the general US population diagnosed as having depressive disorder [40]. The alarm is raised because these results are derived primarily from a sample drawn from people in the community who might have been benefiting from increased and higher levels of social support and services from CBOs, a feature that might distinguish them from the general community of Latina transgender individuals. This could imply that rates of depression in the general population of Latina transgender women may in fact be higher than those in our sample.

Additionally, 32% of our participants indicated that in the two weeks prior to the interviews, they either thought that they would be better off dead or thought of hurting themselves in some way nearly every day or at least on several days. A recent report of the National Transgender Discrimination Survey conducted by the National Center for Transgender Equality and National Gay and Lesbian Task Force indicated that 41% of their transgender respondents reported having attempted suicide compared to 1.6% of the general population [28]. The present sample of Latina male-to-female transgender women who reported more frequent perceived discrimination were more likely to present with severe depression.

Our data show that a large number of the participants experienced discrimination on regular basis. These incidents of discrimination commonly include being accused of being a sex worker and HIV positive; being called names or insulted; being made fun of and treated disrespectfully; and being threatened or harassed. Additionally, being denied employment, housing, and access to public restrooms; being rejected by family members; and being stopped by law enforcement without violating any law were all common examples of discrimination that were mentioned by at least 50% of the participants. These kinds of discrimination can have a profound impact on transgender peoples’ lives [28,41].

A recent meta-analysis of available empirical evidence from studies of other populations shows that discrimination is associated with poorer mental health status in minority populations [42]. Homosexual and bisexual individuals report more lifetime and day-to-day experiences with discrimination than heterosexual individuals and 42% attribute this discrimination to their sexual orientation [29]. Transgender people are significantly more likely than gay men, lesbians, and bisexuals to experience discrimination [18]. Transgender individuals who often feel increasingly isolated, helpless, misunderstood, and discriminated against may become depressed [28]. More research is needed on the social discrimination and isolation specific to Latina transgender women and a closer examination of perceived discrimination and levels of depression. Additionally, research is needed to explore the social network and social support of Latina transgender individuals and investigate whether lack of social support among this segment of our population exacerbates depressive symptoms.

Indeed, a recent study by Nemoto and colleagues [12] assessed the racial/ethnic differences in social support and exposure to violence and transphobia among male-to-female transgender women with a history of sex work and showed that depression was significantly correlated with transphobia, social support and Latina ethnicity. Nemoto and colleagues argue that these findings among Latina transgender individuals need additional study to examine why Latina ethnicity contributed to higher levels of depression. Longitudinal studies that assess the role of social support and the effects of exposure to discrimination and transphobia and its impact on mental health from childhood to adulthood are needed [12].

There was a notable association between self-reported history of sexual partner violence and mistreatment by main partners/spouse/casual partners and severity of depression, even after other related variables (including demographic variables and sex work) were held constant. Almost six out of 10 participants in present study admitted that they had been a victim of violence by a sexual partner. It is not a surprise then that those who were mistreated or victimized by their partners/spouse were almost two times more likely than their counterparts who were not victimized to report severe depression. For transgender people who are abused by their sexual partners, finding help can be very difficult. Undoubtedly, there are not enough appropriate agencies that can provide suitable help. Latina male-to-female transgender women who are victims of sexual partner violence may be particularly reluctant to file complaints because of the discrimination they may face [30]. Although anyone experiencing sexual partner violence may report depressive symptoms [43-46], sexual partner violence within the transgender population calls for further study for the following reasons:

1. Discrimination by law enforcement - Many transgender people have reported being harassed and discriminated against by law enforcement [28,46]. The National Transgender Discrimination Survey indicates that one-fifth of transgender individuals who have interacted with law enforcement reported harassment by police [28]. Moreover, with the Latina transgender population, immigration status may influence whether they seek legal help.

2. Social isolation and rejection - Cases of sexual partner violence are typically characterized by the perpetrator isolating his victim from family and friends despite their efforts to reach out. Given that almost 60% of the sample reported having been a victim of violence by their sexual partner, spouses or casual partners, and 66% of the participants reported that they had been rejected by their families, it is plausible to assume that the degree of isolation experienced by transgender individuals in cases of sexual partner violence may be worse than the level of isolation experienced by other victims of partner violence.

3. Providers - There are not enough providers adequately trained and equipped to meet the needs of the transgender population. The majority of transgender individuals in this study were exposed to sexual violence, and providers need to receive training specifically regarding the signs and symptoms of sexual partner violence in this population. Providers also need to receive training on the specific mental health needs of transgender patients – including addressing depression - in order to comprehensively address their needs.

This study had three main limitations. First, an interpretation of the findings was limited by the cross-sectional nature of the study and an inability to address causation. Second, the data generated were from a non-probability sample, which limits our ability to generalize the findings. Additionally, we used only two items to measure sexual partner violence.

## Conclusions

Latina male-to-female transgender women continue to experience a pervasive pattern of discrimination and depression. An association between level of depression and perceived discrimination was identified. Exposure to discrimination and sexual partner violence leads to increased risk of mental health problems among transgender women. More investigation is needed to determine how to confront these issues to minimize mental health problems. Models investigating perceived discrimination and depression among the transgender population should include sexual partner violence as a potential confounding variable.

## Competing interests

The authors declare that they have no competing interests.

## Authors' contributions

MB carried out the study design, data analysis and prepared the first draft of the manuscript. FG participated in designing the study, the analysis and interpretation of the data, and revising the manuscript. Both authors read and approved the final manuscript.

## Pre-publication history

The pre-publication history for this paper can be accessed here:

http://www.biomedcentral.com/1471-2458/12/663/prepub
